# Acupuncture De-qi: From Characterization to Underlying Mechanism

**DOI:** 10.1155/2013/518784

**Published:** 2013-09-08

**Authors:** Shi-Peng Zhu, Li Luo, Ling Zhang, Song-Xi Shen, Xiao-Xuan Ren, Meng-Wei Guo, Jia-Min Yang, Xiao-Yu Shen, Yong-Si Xu, Bo Ji, Jiang Zhu, Xiao-Hong Li, Lu-Fen Zhang

**Affiliations:** School of Acupuncture-Moxibustion and Tuina, Beijing University of Chinese Medicine, Beijing 100029, China

## Abstract

De-qi refers to the participant's subjective sensations and objective body responses as well as the acupuncturist's perceptions while the acupuncturist needles certain acupoints in the participant's body. In recent years, De-qi is getting increasing attention of the researchers and many efforts have been made to understand its mechanism. By the broad literature survey, this paper explores the subjective De-qi sensation of the patients, its influencing factors, and the resulting physiological responses. The purpose of this paper is expected to find out a possible mechanism of De-qi and to provide certain scientific evidence for acupuncture fundamental research and clinical practice.

## 1. Introduction 

De-qi, originated from *Neijing* (*Internal Classic*), is regarded as one of the most important principles and the key to the successful acupuncture treatment since it is related to clinical efficacy [1–3]. In recent years, researches in this field mainly focus on qualitative and quantitative evaluations of De-qi by scales, influencing factors of De-qi and physiological responses aroused by De-qi. The mechanism of acupuncture De-qi is still not well explained although a little progress has been made. 

## 2. Study on the Subjective De-qi Sensations 

Subjective De-qi sensations refer to participants' subjective feelings triggered by De-qi during needling and needle sensation of the acupuncturist. Nowadays, it is mainly evaluated by the scale survey, including questionnaires based on the feeling of patients and opinions of experienced acupuncturists. Many scales include both qualitative description and quantitative evaluation (e.g., VAS score) of De-qi. 

In Vincent and his colleagues' study, the McGill Pain Questionnaire (MPQ) was modified by 10 experienced acupuncturists into a final scale that consists of 20 items concerning pain and needle sensations and was applied to 125 participants who received acupuncture. A principal component analysis identified 7 factors, and the first was a composite of dull-heavy sensations which included pulling, numbness, heaviness, dullness and aching. These feelings were very similar to the description of De-qi sensations in TCM [4]. However, all the sensations in the scale were primarily drawn from a pain questionnaire which was not focused specifically on De-qi. This could mislead patients to use painful sensations to describe De-qi. In a qualitative study of De-qi, 29 experienced acupuncturists separated 25 sensations into two clusters: one associated with De-qi and another with acute pain. De-qi cluster included aching, dull, heavy, numb, radiating, spreading, and tingling sensations, while the cluster associated with acute pain contained hot burning, hurting, pinching, pricking, sharp, shocking, stinging, and tender sensations [5]. However, this study did not involve patients' perceptions during acupuncture. Kim et al. studied the real-life patient's experience of insertion, manipulation, and retention stages of acupuncture through in-depth interview, group discussion, and expert panel assessment for content validity. The result indicated that “refreshing or relieving,” “dull,” “tingling,” “painful,” and “electrical shock” sensations were how patients feel throughout the whole phases [6].

In order to develop a sensation questionnaire that was able to discriminate between pain and De-qi, White et al. [7] developed the Southampton Needle Sensation Questionnaire which was designed following qualitative interviews with patients, literature review, and consultation with experts. The questionnaire was then piloted and validated in 227 patients. Two clusters of needling sensations were demonstrated in the final questionnaire, namely, “Aching De-qi” (De-qi with pain) and “Tingling De-qi” (De-qi only). The former included deep ache, dull ache, discomfort, heaviness, pressure, and bruised and stinging pain, while the latter contained tingling, warm, spreading, fading, numb, twinge, and throbbing sensations. In addition, they found that among 17 items in the final questionnaire only “sharp pain” had a significant partial correlation with the pain VAS score, so it should not be considered as an indicator of De-qi sensation. Kong et al. [8] developed a subjective acupuncture sensation scale (SASS) which contains stabbing, throbbing, tingling, burning, heaviness, fullness, numbness, soreness, and aching when they launched a pilot study on acupuncture analgesia. In their clinical practice with the scale, they found that numbness and soreness induced by acupuncture had a significant correlation with analgesia, which indicated that De-qi had a certain relationship with curative effect. After years' of application of the scale, “stabbing” and “burning” sensations were replaced by “sharp pain,” “deep pressure,” “dull pain,” “warmth,” and “cold.” The modified scale was called “MGH Acupuncture Sensation Scale” (MASS). When translating MASS into Chinese version, researchers removed “sharp pain” from the scale because it is not commonly regarded as a kind of De-qi sensation. However, overall validity and reliability of the modified scale were not much affected [9].

In a study with the purpose of describing Chinese patients' acupuncture experience, the most common types of needling sensations reported by 200 subjects were “distension” (94%), “soreness” (81%), “electric shock” (81%), and “numbness” (78%). 82% of the subjects believed that needling sensation was very important for acupuncture treatment and 68% further indicated that the stronger the needling sensation was, the more effective the therapy would be. Meanwhile, 81% of subjects regarded needling sensation as a very comfortable and relaxing sensation. The result also showed that the needling sensation had a migratory nature. 89% of subjects reported that the needling sensation travelled away from the puncturing points or travelled among the needling points. However, the duration and intensity of migratory sensation differed among patients [10]. Hui et al. [11] investigated the perception of De-qi of Chinese and American acupuncturists. The result indicated that 47 out of 86 acupuncturists agreed that dull pain was considered De-qi and over half believed that it was beneficial, while sharp pain was not De-qi and harmful. They also found that Chinese patients enjoy De-qi experience, whereas those in the US did not.

Different subjective De-qi sensations actually indicating different nerve fibers have been activated. Wang et al. found that numbness was conveyed mainly by A*β*/*γ* fibers, distention and heaviness by A delta fibers, and soreness by C fibers [12]. Beissner et al. showed that pricking sensation was closely linked to A delta fibers, while dull and pressing sensations were related to C fibers [13]. Furthermore, these fibers attach to different areas of the brain. Thus, different De-qi sensations may trigger different brain reactions based on the types of afferent nerve fibers involved and finally lead to various therapeutic effects. For example, Kong et al. [14] demonstrated that there was a significant correlation of analgesia with SASS ratings of numbness and soreness, but not with stabbing, throbbing, tingling, burning, heaviness, fullness, or aching when they needled on LI4, ST36, and SP6.

According to the researches mentioned above, it could be concluded that perception of De-qi by patients are consistent with that described in Traditional Chinese Medicine, such as “sore”, “numb”, “distended”, “heavy”, “electric”, and “warm” “cold” sensation. It is a special sensation between pleasant and outright pain, or tolerable dull pain for patients, therefore, sharp pain should not be included. De-qi sensation is also a sign to acupuncturists that indicates the curative effect has been initiated. Most of the experienced acupuncturists and patients believe that De-qi has a significant impact on curative effect. 

## 3. Influencing Factors of De-qi 

### 3.1. Specificity of Acupoints

Specificity of acupoints is one of the influencing factors of De-qi. In a study that applied EA on paired acupoints of ST36-ST28 and CV4-CV12, significant differences of De-qi intensity were found in soreness, fullness, and heaviness between ST36-ST28 and in fullness between CV4-CV12 [15]. Acupoints in these two groups share the same meridian category and tissue structure, but vary in nerve innervations: ST36 is innervated by L3/4 while ST28 by T12. Therefore, nerve innervations of acupoints have a significant influence on De-qi.

Although De-qi is affected by nerve innervations, researchers found that there was no correlation between direct stimulation to nerves around the acupoints and obtaining De-qi sensation. In an ultrasound imaging study, Streitberger et al. [16] found no association between the number of nerve contacts and De-qi when applying acupuncture on PC6. In some cases, De-qi sensation had not been elicited even when the needle was inserted into the nerve. Similar studies found that De-qi sensation was well achieved before the needle touched the median nerve under PC6, suggesting that irritation of the nerve was not directly involved in generating it [17]. Therefore, De-qi should be a physiological phenomenon triggered by both the central and peripheral nerve systems rather than a simple reaction to direct neural stimulation. 

Although De-qi sensation could be elicited at both acupoints and nonacupoints, the subsequent physiological responses are different. Feng et al. [18] found that after needling ST36 with De-qi, the limbic/paralimbic regions such as amygdale, hippocampus, and anterior cingulated gyrus emerged as network hubs. However, this trend did not occur when needling nonacupoint nearby, although De-qi sensation was also elicited. This result indicated that the effect of De-qi on central nerve system might be based on the function of acupoints. Wei et al. [19] investigated the effect of EA at LI4 and non-acupoint on blood flow in the middle cerebral artery. It demonstrated that there were no significant differences in De-qi intensity between LI4 and non-acupoint, but the application of EA at LI4 caused a significant decrease in ipsilateral blood flow velocity (BFV) and further decrease in the poststimulation period, EA at non-acupoint did not alter the value of both BFVs during the stimulation period. It only caused significant decrease in both BFVs during the poststimulation period. 

The above studies showed that acupoints and non-acupoints might induce different central responses; even the same De-qi sensation was elicited by the same needling techniques. Therefore, the specificity of acupoints plays an important role in De-qi's effects on physiological function.

### 3.2. Acupuncture Manipulation

Many researches proved that acupuncture manipulation had a significant impact on De-qi. Rotation is the most commonly used manipulation for eliciting De-qi. Benham et al. [20] found that De-qi VAS scores were higher during deep needling (15–25 mm) on ST36 with bidirectional rotation when compared to superficial (5 mm) needling with mock bi-directional rotation in the sensations of stabbing, tingling, heaviness, soreness, and aching. Park et al. [21] demonstrated that the introduction of needle rotation significantly increased deep, dull, and heavy sensations, but not pricking and sharp sensations. Another study indicated that rotating the needle in real acupoints (LR3 and GB40) with De-qi activated secondary somatosensory cortex bilaterally, the left frontal lobe, the right side of the thalamus, and the left side of cerebellum more significantly than those without rotation, but this trend did not occur in non-acupoints [22]. This result showed that acupuncture with needle rotation could activate certain parts of the brain, but this effect could be achieved by stimulating on acupoints.

 “Needle grasp” sensation felt by acupuncturist during needling is an important indicator of De-qi. It has been described in* Biao you fu (Song to Elucidate Mysteries) as follows*: “the arrival of qi like a fish biting on a fishing line.” Langevin et al. [23] believed that connective tissue is the foundation of grasp sensation from the aspect of mechanical signaling. Their study observed that needle rotation was accompanied by marked thickening of the SC connective tissue layer in the area surrounding the needle. Winding of tissue around the needle leads to the generation of a mechanical signal by the pulling of collagen fibers and matrix deformation during rotation. Then, the signal was transmitted into cells and caused the subsequent downstream effects. This may be the mechanism of the Meridian qi migratory. A similar study pointed out that the pull-out force was significantly greater with needle rotation than that without rotation because of the winding of tissues around the needle [24]. This result suggested that the pull-out force could be used as an indicator that evaluates De-qi quantitatively.

The depth of needling may also affect De-qi sensation. In an ultrasound guided study, Park et al. [21] demonstrated that pricking and sharp sensations were more frequently received when pressed but not inserted into the skin surface and needled at the lower bolder of dermis. Deep, dull, heavy, spreading, and electric shock sensations occurred when the needle was inserted 2–15 mm beyond the first perimuscular fascia. In a study that measures participants' De-qi sensation and pain threshold by comparing superficial needling and deep needling with and without rotation on SP6, SP9, ST36, and GB39, Choi et al. [25] found that deep needling with bi-directional rotation had a marked effect in increasing both De-qi sensation and pain threshold, which is better than that of deep needling without rotation or superficial needling. Therefore, the depth of needling and the rotation manipulation have a cooperative effect in generating De-qi sensation and curative effects.

### 3.3. Methods of Stimulation

Stimulation method is another factor that affects De-qi. Some studies compared the different subjective De-qi sensations and physiological responses caused by manual acupuncture and EA. Kong et al. [26] demonstrated that the sensations of manual acupuncture on LI4 were mainly soreness and distension, while those of EA were mainly tingling and numbness. Their research also found that fMRI signal increased in precentral gyrus, postcentralgyrus/inferior parietal lobule, and insula during EA while decreased in posterior cingulated, superior temporal gyrus and insula during manual needling manipulation. Therefore, different brain mechanisms may be involved during different methods of stimulation. Leung et al. [27] proved that De-qi sensations were qualitatively and quantitatively different between manual and electrical acupunctures. De-qi VAS score significantly increased after EA on LI4 in comparison to manual acupuncture. The most predominant De-qi sensation with EA was tingling, whereas in the manual acupuncture aching was the most predominant one. When studying the impact of manual acupuncture and tactile stimulation on De-qi, Hui et al. [28] found that manual acupuncture on LR3, LI4, and ST36 could elicit higher frequency and intensity of De-qi sensation than tactile stimulation. The most significant difference between two methods lies in aching, soreness, pressure, and dull pain.

### 3.4. Psychological Factors

Some believe that De-qi might be a central phenomenon of awareness and consciousness because they found that sham laser acupuncture, which was inactivated and had no cutaneous sensory input, could elicit the same De-qi sensation as verum laser acupuncture at LI4, LU7, and LR3 with regard to frequency, intensity, and quality [29]. However, a great many researches pointed out that De-qi is a kind of physiological response triggered by acupuncture and has less relativity with psychological factors. Xiong et al. [30] explored the relationship among De-qi, psychological factors, and clinical efficacy when treating primary dysmenorrhea by needling on ST36, SP6, and CV3. Psychological factors of patients including belief in acupuncture, the level of nervousness, anxiety, and depression were quantitatively assessed. The result showed that psychological factors contributed little to De-qi and the correlation between De-qi and therapeutic efficacy was greater than that between psychological factors and clinical efficacy. 

Many studies also showed patients' expectancy of De-qi sensation or their acupuncture experience did not influence what they acutely experienced during needling significantly. In Park and colleagues' study [31], the expected sensations of 38 acupuncture naive female volunteers were penetrating, tingling, pricking, and burning. However, they experienced aching, pulling, heavy, dull, electric, and throbbing sensations when the needling was done on LI4. Their study between acupuncture experienced and naive also supported that previous experience did not affect people's expectation and would not hinder people from experiencing De-qi [32]. Studies mentioned above showed that psychological factors might have certain influence on De-qi, but it is not the decisive factor.

In conclusion, the specificity of acupoints, needling manipulation, and methods of stimulation are key factors that affect De-qi, whereas psychological factors have comparatively less influence on it. Specificity of acupoints may be the intrinsic factor of De-qi, which could be elicited by external factors like needling manipulation and methods of stimulation. De-qi is most likely to be elicited when needling acupoints in appropriate depth with certain manipulations.

## 4. Objective Response to De-qi

### 4.1. Autonomic Response to De-qi

Autonomic response refers to a series of physiological changes of the body triggered by external stimuli, mainly including changes of skin conductance, heart rate, blood pressure, and blood flow. These changes, with an immediate presence, are controlled by autonomic nervous system. Abnormality of autonomic function, such as sympathetic nerves overactivation, has a relation with chronic pain in certain diseases [33, 34]. Researches show that acupuncture De-qi could regulate autonomic responses by balancing sympathetic nerve and parasympathetic nerve activities.

Sakai et al. [35] found that acupuncture on right trapezius muscle with De-qi suppressed sympathetic nervous activity and stimulated parasympathetic nervous activity, manifested as reduced heart rate and the ratio of low frequency components to high frequency components of heart rate variability (index of sympathetic activity) and increased systolic pressure. Thus, acupuncture De-qi may play its analgesia role by inhibiting sympathetic and exciting parasympathetic nerve activity. Acupuncture De-qi also has influence on blood flow and skin temperature around acupoints. Kuo et al. [36] found that acupuncture on LI-4 could cause stress reaction of parasympathetic nerve, which raised skin temperature of the palm through cutaneous vessels vasodilatation and increased blood flow of LI-11 when De-qi (soreness and numbness sensations) occurred. Therefore, changes of blood flow and skin temperature may be the mechanism through which certain De-qi sensations (e.g., soreness and numbness) occurred. Sandberg et al. [37] studied the influence of different stimulations (ST36 superficial puncture, muscle puncture without De-qi, and muscle puncture with De-qi) on participants' blood flow of the skin and muscle. They found that muscle blood flow increased following both muscle puncture without De-qi and muscle puncture with De-qi, with the latter being more obvious in the initial 5 min. Besides, skin blood flow also increased for 5 min following De-qi. On the contrary, no increase was found following superficial puncture.

### 4.2. Effects of De-qi on Electroencephalogram

Electroencephalogram (EEG) is another objective indicator of De-qi and its change is significantly associated with autonomic nervous functions. Yin et al. [38] demonstrated that participants with higher De-qi sensation during needle retention showed significant changes in alpha band powers over the periods before, during, and after needle retention while those with lower De-qi sensations did not. The result indicated a good correlation between EEG changes and De-qi sensation. Sakai et al. [35] showed that De-qi induced by acupuncture manipulation not only caused autonomic changes (HR and SBP), but also nonspecifically increased the power of all spectral bands except the gamma band of EEG and the correlation between De-qi and EEG changes was good. Besides, their preliminary result reported that acupuncture without De-qi sensation induced much less effects on EEGs and autonomic functions. Tanaka et al. [39] reported that acupuncture increases all bands of EEG power, and this phenomenon occurred only with autonomic changes triggered by acupuncture. Previous studies also showed that autonomic nerve functions were closely related to EEG changes [40]. Therefore, autonomic changes induced by acupuncture with De-qi are probably mediated through the central nervous system. Changes in EEG are related to specific De-qi sensation induced by acupuncture manipulation, but not to a general arousal state.

### 4.3. Effect of De-qi on Limbic-Paralimbic System and Subcortical Structure of the Brain

Several studies observed the impact of De-qi on brain fMRI blood oxygen level-dependent (BOLD) signals and found out that acupuncture needle sensations of De-qi without sharp pain and De-qi with sharp pain are associated with different patterns of fMRI BOLD signal activation and deactivation in limbic-paralimbic system and subcortical structure of the brain.

 Fang et al. [41] found that acupuncture produced extensive deactivation of the limbic-paralimbic-neocortical system. In their study, amygdale (acupoints LV2 and ST40) and hippocampus (acupoints LR2, LR3, and ST44) showed a fMRI BOLD signal deactivation in participants who experienced De-qi without sharp pain. Asghar et al. [42] reported different fMRI BOLD signals of brain between De-qi and acute pain when needling at LI4. Predominately, De-qi sensation deactivated the BOLD signals in the limbic/subcortical structure and the cerebellum, whereas acute pain sensation (De-qi > pain contrast) increased the signals in these areas. Hui et al. [43] showed a fMRI BOLD signal decrease in limbic and paralimbic structures of cortical and subcortical regions in the telencephalon, diencephalon, brainstem, and cerebellum when participants experienced De-qi sensation only (including dull pain but not sharp pain) at ST36. However, when De-qi sensation was mixed with sharp pain, the hemodynamic response was mixed, showing a predominate signal increase.

In general, predominant De-qi sensation deactivated the fMRI BOLD signals in the limbic-paralimbic system and the subcortical structure of the brain, while the sharp sensation activated the BOLD signals in these regions. The decreases in fMRI BOLD signals are not clearly explained yet, but increasing clues point to that it is a manifestation of neuronal deactivation [44]. Limbic-paralimbic system and neocortical structure play an important role in processing the cognitive and affective dimensions of pain signals and act as the regulatory center of emotion, cognition, consciousness, autonomic, endocrine, and immunological functions. Therefore, it is possible that acupuncture De-qi performs its comprehensive function by inhibiting neuronal activity in the limbic-paralimbic system and the subcortical structure of brain. 

## 5. Discussion

Nowadays, the studies on subjective De-qi sensation are mainly based on the scale survey, including both qualitative description and quantitative evaluation. These studies found that De-qi sensation is a special sensation between pleasant and outright pain. The most common perception of De-qi sensation by patients is consistent with that described in TCM, such as “sore,” “numb,” “distended,” “heavy,” “electric,” and “warm” sensations. Different De-qi sensations may trigger different brain reactions based on the types of afferent nerve fibers involved and finally lead to various therapeutic effects. It should be noted that there are some limitations in the existing scales. In TCM theory, De-qi sensation felt by acupuncture practitioner is even more important than that felt by patients. Park et al. [45] also found that needle sensations of practitioners are more objective than those of patients. However, most of the scales did not take practitioners' needle sensations into consideration. 

Many studies proved that specificity of acupoints, needling manipulations, and stimulation methods are key affecting factors of De-qi, while the psychological factor has relatively less influence. It is found that De-qi is most likely to be elicited when needling acupoints in appropriate depth with certain manipulation. The acupuncture practitioners are vital to elicit De-qi sensation because they locate the acupoints and conduct all the manipulations. Therefore, the background of the practitioner such as their experiences in acupuncture practice should be taken into account in future studies on influencing factors of De-qi.

De-qi could trigger a series of objective changes from the center to the periphery, including changes of autonomic functions such as HR, blood flow, skin temperature, and deactivation of fMRI blood oxygen level-dependent (BOLD) signals in the limbic/paralimbic system and the subcortical structures of the brain as well as EEG changes. Increasing clues point to the fact that deactivation of fMRI BOLD signals is a manifestation of neuronal deactivation. Therefore, it is possible that acupuncture with De-qi obtains its curative effects by affecting autonomic functions through the central nervous system and regulating neuronal activity of the limbic-paralimbic system and subcortical structure of the brain. A series of cascade reactions may be activated to ultimately restore the homeostasis inside the patients' body by regulating nerve-endocrine-immune network as a whole after De-qi is well achieved. However, most of the researches on acupuncture De-qi that we mentioned were carried out on healthy subjects but not on patients. Subjects in diseased condition will respond differently in comparison with healthy subjects. For example, Liu et al. [46] reported that during acupuncture both hypothalamus response and De-qi score were different between heroin addicts and healthy subjects. Compared to healthy subjects, the De-qi score of heroin addicts was significantly higher and the activation of hypothalamus was more robust. In *Neijing* (*Internal Classic*), it is described that people with excess constitution can obtain De-qi easily, while people in deficient state may hardly experience it. Therefore, subjects in diseased conditions should be observed in future De-qi researches.

## 6. Conclusion

As an important component of acupuncture, De-qi is getting increasing attention of the researchers. Current researches partly revealed the underlying mechanism of De-qi from the aspect of subjective De-qi sensations, its influencing factor, and the resulting physiological responses. Further researches, particularly on De-qi in diseased condition, are needed in order to understand De-qi more objectively and comprehensively.
